# Impact of Physical Activity and Weight Loss on Fat Mass, Glucose Metabolism, and Inflammation in Older African Americans with Osteoarthritis

**DOI:** 10.3390/nu12113299

**Published:** 2020-10-28

**Authors:** Andrew McLeod, Linda Schiffer, Karla Castellanos, Andrew DeMott, Sarah Olender, Marian Fitzgibbon, Susan Hughes, Giamila Fantuzzi, Lisa Tussing-Humphreys

**Affiliations:** 1 Department of Kinesiology and Nutrition, University of Illinois at Chicago, Chicago, IL 60612, USA; amcleo2@uic.edu (A.M.); giamila@uic.edu (G.F.); 2 Institute for Health Research and Policy, University of Illinois at Chicago, Chicago, IL 60608, USA; lschiff@uic.edu (L.S.); ademot1@uic.edu (A.D.); mlf@uic.edu (M.F.); shughes@uic.edu (S.H.); 3 Department of Medicine, University of Illinois at Chicago, Chicago, IL 60612, USA; kcaste5@uic.edu (K.C.); solender10@gmail.com (S.O.); 4 Center for Research on Health and Aging, Institute for Health Research and Policy, University of Illinois at Chicago, Chicago, IL 60608, USA; 5 Department of Pediatrics, University of Illinois at Chicago, Chicago, IL 60612, USA; 6 University of Illinois Cancer Center, University of Illinois at Chicago, Chicago, IL 60612, USA; 7 Department of Community Health Sciences, School of Public Health, University of Illinois at Chicago, Chicago, IL 60612, USA

**Keywords:** cancer prevention, adiposity, insulin resistance, physical activity, dietary intervention, African American

## Abstract

(1) Background: There are currently very few interventions performed within a community setting that compare the effects of physical activity (PA) versus PA plus weight loss on cancer and chronic disease risk in older African Americans. Therefore, we investigated the impact of an 8 week (24 session) PA intervention compared to a PA plus weight loss intervention on fat mass, glucose metabolism, and markers of inflammation in older, overweight and obese African Americans. (2) Methods: Subjects were randomized to a PA (*n =* 83) or PA plus weight loss (*n =* 72) intervention that met three times weekly for 8 weeks. At baseline and post-intervention, anthropometrics, body composition, systemic inflammation (high-sensitivity C-reactive protein, tumor necrosis factor-α, and interleukin 6), fasting glucose, insulin and homeostasis model assessment-insulin resistance (HOMA-IR) were determined. (3) Results: Subjects had a mean age of 67 years (SD = 5.3) and were mostly women (88%). The PA plus weight loss group lost more total and visceral fat than the PA group (−4.0% vs. +0.6% and −4.1% vs. +3.7%, respectively, *p* < 0.01 for both). Changes in inflammation and glucose metabolism were similar between groups post-intervention. Within the PA plus weight loss group only, serum insulin and HOMA-IR decreased significantly. (4) Conclusions: PA combined with weight loss can decrease total and visceral fat mass and improve insulin sensitivity, confirming that these cancer- and chronic disease-related risk factors are influenced by relatively modest lifestyle changes in the short term.

## 1. Introduction

The United States population is at high risk for cancer and several chronic diseases, such as cardiovascular disease (CVD) and type 2 diabetes (T2D). Approximately 42% of the United States population is predicted to develop cancer over their lifetime [1], at least 56% CVD [2], and at least 33% T2D [3]. Obesity and low levels of physical activity (PA) are risk factors for several types of cancer, such as breast and colorectal cancers [4,5,6,7,8,9], and for several other non-communicable diseases, such as CVD [10,11], and T2D [12]. These risk factors are highly prevalent in African American (AA) women, with those 20 years and older having the highest rates of obesity and those aged 45–64 engaging in less PA than their non-Hispanic white counterparts [13,14]. The high prevalence of these risk factors may explain why, compared to non-Hispanic whites aged 55–79 years, AAs experience higher rates of esophageal, renal, colorectal, and advanced uterine cancers [15,16]. AAs, especially women, also have higher chronic disease risk such as for CVD [17] and T2D [18].

Obesity increases cancer, CVD, and T2D risk through several likely mechanisms, including a low-grade chronic pro-inflammatory response and glucose dysmetabolism [19,20,21]. Often accompanying obesity is an increased amount of circulating pro-inflammatory cytokines, such as interleukin-6 (IL-6), which can stimulate upregulation of anti-apoptotic genes [22,23]. Another of these pro-inflammatory cytokines is tumor necrosis factor-alpha (TNF-α), which enhances tissue proliferation and cancer metastasis [19,24]. The pro-inflammatory capacity of these cytokines also can lead to glucose dysmetabolism, characterized by hyperinsulinemia and hyperglycemia, both of which can promote carcinogenesis [19,25], CVD [20], and T2D [21].

Low levels of PA are associated with increased risk of cancer, CVD, and T2D through inflammation and glucose dysmetabolism as well [26,27,28]. Several cross-sectional studies show that, compared to adults who engage in higher levels of PA, those engaged in lower levels of PA have increased levels of the acute phase protein, C-reactive protein (CRP), and higher levels of IL-6, and TNF-α [29,30,31,32,33,34]. For example, Lamonte and colleagues showed that, among 4832 older, overweight, and ethnically diverse women, decreased PA measured objectively by accelerometry was associated with increased levels of CRP [32]. Given that inflammation is mechanistically linked to cancer and CVD, a decrease in PA may lead to increased cancer and CVD risk.

Numerous studies have also shown that decreased PA is associated with altered glucose metabolism. Lamonte and colleagues showed that decreased PA was associated with increased blood glucose and insulin levels [32]. In another study, McGlory and colleagues demonstrated that older, overweight adults who voluntarily decreased PA for one week developed insulin resistance that failed to resolve after two weeks of subsequent PA [35]. Given that glucose dysmetabolism is mechanistically linked to cancer and T2D, a decrease in PA may lead to increased cancer and T2D risk.

Obesity and low levels of PA may lead to cancer and chronic disease, suggesting that weight loss and an increase in PA may help prevent them. Studies show that, in older populations, weight loss and PA interventions result in reductions in CRP and pro-inflammatory biomarkers, such as TNF-α, IL-6, and IL-8 [36,37,38,39,40,41]. Furthermore, reductions in IL-6 and CRP have been significantly correlated with reduction in visceral adipose tissue (VAT) volume and total body fat mass [38]. Similar interventions have also led to reductions in blood glucose levels, improvements in insulin sensitivity, and decreased insulin levels in older populations [40,42,43]. Some interventions, however, have not resulted in decreased glucose levels [44,45]. This discrepancy may be due to the lower intensity of exercise in these studies compared to those in which glucose levels improved.

Despite the reported evidence that PA or PA plus weight loss can lower inflammation and improve glucose metabolism, only one randomized controlled trial has tested such interventions in predominately older AAs [46]. Many other trials have involved middle-aged adults and/or involved a majority of white participants [36,37,38,39,41,42,44,45,47,48,49,50,51,52,53,54,55,56,57]. Furthermore, to our knowledge, only two other studies have tested such interventions in a “real-world” setting—one in which the interventions are kept low cost and designed to be easily implemented into existing community structures, such as programming within local park districts [44,45]. Consequently, we tested the effect of PA alone (Fit and Strong! (F&S!)) or in combination with weight loss (Fit and Strong! Plus (F&S!+)) among older, obese AAs on cancer, CVD, and T2D risk-related biomarkers, including markers of inflammation and glucose metabolism. We hypothesized that these biomarkers of cancer and chronic disease risk would decrease significantly in both groups, but more so in participants randomized to F&S!+, owing to its greater emphasis on weight loss. Furthermore, given the strong connection between adipose tissue, inflammation and glucose dysmetabolism, we predicted that a reduction in total fat mass and VAT would correlate with a reduction in chronic disease-risk biomarkers, irrespective of overall weight loss.

## 2. Materials and Methods

### 2.1. Recruitment Strategy and Inclusion/Exclusion Criteria

This study is ancillary to a larger study that investigated the effectiveness of F&S!+, compared to F&S!, in reducing weight and improving diet quality, PA, osteoarthritis (OA) symptoms, anxiety, and depression among a cohort of 413 overweight and obese adults over the age of 60 years with self-reported lower-extremity (LE) OA residing primarily in Chicago, Illinois. (Grant # R01AG039374, NCT # 03180008) [58].) The interventions were conducted in Chicago Park District sites and local churches. Recruitment consisted of flyer distribution at local businesses, churches, and senior residences, and emails to subscribers of the Arthritis Foundation’s listserv. Interested persons were screened over the phone for both the parent and current study using the eligibility criteria below.

Inclusion criteria for the parent study stipulated that participants have LE OA determined by pain in or around at least one knee or hip most days in the past month, or pain or stiffness in or around hips, knees, ankles, feet, or lower back on most days of at least one month of the last six months; age 60 years and older; no current participation in a regular PA program and completing less than 150 min of combined moderate and vigorous PA per week; calculated body mass index (BMI) of 25–50 kg/m^2^; and ability to attend class at specified times and to participate in measurement and intervention procedures. Exclusion criteria included a score of three or more on the 10-item Mental Status Questionnaire [59]; uncomplicated hip or knee surgery within the previous six months or surgery with complications within the past year; plans for hip or knee surgery within the next year; steroid injections in either knee or hip within the previous three months; diagnosis of rheumatoid arthritis; uncontrolled diabetes, or health conditions that might interfere with exercise. The Exercise and Screening for You (EASY) screener [60] was used to identify health contraindications to exercise. Participants who reported one or more high-risk conditions on the EASY screener were required to obtain a physician’s approval before participating. 

The ancillary study (American Cancer Society of Illinois, #261775) had the same inclusion criteria. In addition, participants had to self-report a body weight of 450 pounds or less, be cancer free for the previous five years, and self-describe as AA. Exclusion criteria were the same. A subsample of participants (*n =* 155) from the parent study participated in the ancillary study procedures (i.e., blood draw, and body composition via dual energy X-ray absorptiometry (DXA)).

The University of Illinois at Chicago (UIC) Institutional Review Board reviewed and approved both the parent and ancillary study (2012-0277; 2013-0098). Prior to enrolling in the parent and ancillary study, participants provided informed, written consent.

### 2.2. Parent Study Interventions

A full description of the parent study design and methods is reported elsewhere [58]. Briefly, F&S! is an evidence-based PA program which the Centers for Disease Control and Prevention and National Council on Aging recommend for older adults with OA [61]. The program seeks to reduce the number and severity of OA-related symptoms through strengthening of muscle and bone. It also aims to promote behavior change by reinforcing self-efficacy for exercise and exercise adherence. Certified fitness instructors carry out these aims in thrice weekly 90 min sessions for eight consecutive weeks. Eight weeks was chosen to keep costs lower compared to most other interventions which last at least 16 weeks. This makes the intervention easy to disseminate, both by simplifying training of staff and by avoiding participant burnout. At each session, a certified fitness instructor focused on flexibility/balance, aerobics, and lower-extremity strengthening exercises for twenty minutes each. For thirty minutes afterward, the instructor facilitated a group discussion on, and used a structured curriculum to provide education about, OA symptom management.

F&S!+ is the same as F&S! in many components but not all. The instructor-led sessions of F&S! related to OA symptom management are condensed to facilitate the addition of sixteen sessions focusing on calorie reduction and improving diet quality to help participants lose weight. Emphasis is also placed on weight loss self-efficacy through the use of food diaries and weekly weigh ins, which help participants track positive changes. Ultimately, F&S!+ aims to promote PA, healthy eating behaviors, self-efficacy associated with both, and 5% weight loss within 6 months.

### 2.3. Study Measures and Data Collections Methods

Data were collected from participants at the beginning of the intervention and at eight weeks. At baseline and follow up, ancillary study participants completed surveys and physical assessments and underwent a whole-body DXA scan and a fasting venous blood draw. Participants also refrained from vigorous exercise for 24 h and refrained from non-steroidal anti-inflammatory drugs, insulin, oral hypoglycemic agents, and dietary supplements. They wore metal-free clothing (for DXA scan) and had to be cold and flu free for at least seven days preceding data collection.

Demographic information was collected at baseline with a standard interviewer-administered questionnaire. A health history questionnaire was given to record self-reported disease status and medication usage. Trained research staff measured height in duplicate at baseline to the nearest 0.1 cm with a stadiometer and measured weight in duplicate at both visits to the nearest 0.1 kg with a digital scale. Waist circumference was measured just above the iliac crest at the mid-axillary line [62] to the nearest 0.1 cm, using a Gulick 150 cm anthropometric tape. Body composition, including total fat mass, estimated VAT and lean mass, was measured via DXA. If a participant was too large to have a whole-body DXA scan, a half scan was completed and whole-body composition was calculated from the half scan.

Fasting venous blood samples were processed for serum, aliquoted, and stored at −80 °C until analysis. Systemic inflammation was measured through CRP, IL-6, and TNF-α. High-sensitivity CRP was analyzed at Quest Diagnostics (Wood Dale, IL, USA) via nephelometry. IL-6 was analyzed at UIC with Quantikine^®^ HS Human Immunoassay for IL-6 (R&D Systems, Minneapolis, MN, USA). TNF-α was analyzed via ELISA using the Quantikine Human TNF-α Immunoassay (R&D Systems, Minneapolis, MN, USA). Glucose metabolism was measured through fasting serum glucose and fasting serum insulin. Fasting serum glucose was measured via spectrophotometry and fasting serum insulin via immunoassay at Quest Diagnostics (Wood Dale, IL, USA). Insulin resistance was calculated from the homeostatic assessment—insulin resistance (HOMA-IR) model equation [HOMA-IR = fasting insulin (mU/L) × fasting glucose (mmol/L)/22.5] [63].

### 2.4. Power and Statistical Analysis

For this ancillary study, we performed an a priori power analysis using PASS software (2008, Kaysville, UT, USA) from published data. Group sample sizes of 71 in customary F&S! and 71 in F&S!+ could provide 80% power to detect moderate effect sizes (0.47–0.48) for the pro-inflammatory cytokines (CRP, IL-6, and TNF-α). With an estimated 5% attrition rate from formative work, 150 subjects were recruited (75/group) to achieve adequate power to detect the effect sizes [36,41].

Data were collected via paper-based questionnaire and entered into a Research Electronic Capture (REDCap) database. Prior to statistical analysis, data entry errors and the distribution of variables were assessed. Because of their non-normal distribution, CRP, IL-6, TNF-α, glucose, insulin and HOMA-IR were log transformed for analysis. Because we used a subset of a larger randomized sample, differences by treatment group at baseline were assessed via t-test, chi-square test or a non-parametric equivalent. Between- and within-group changes from baseline to post-intervention, adjusted for baseline BMI, gender, age, and intervention site/iteration, were assessed using generalized estimating equations (GEE), a method that accounts for intra-individual correlation over time [64]. The weight and BMI models did not include baseline BMI as a covariate, since the baseline value was included in the outcome vector. Adjusted baseline and post-intervention mean and geometric mean were also estimated from these models. Spearman rank correlations were used to examine relationships between changes in biomarkers (insulin, glucose, TNF-α, IL-6, and CRP) and changes in VAT mass and total fat mass, adjusted for change in body weight for the total sample combined. The statistical significance of these correlations was assessed with and without correction for multiple testing (*p* < 0.005 or *p* < 0.05). For other analyses, *p*-values of <0.05 were considered statistically significant. All analyses were conducted with SAS software (version 9.4, SAS Institute, Cary, NC, USA).

## 3. Results

Table 1 shows demographic and health characteristics of participants included in the ancillary study (*n =* 155). The majority were 60 to 69 years of age, female, retired, and had at least some college experience. There were no differences at baseline between groups for any of the demographic variables.

Table 2 shows anthropometric and body composition means at baseline and post-intervention adjusted for gender, age, iteration, and baseline BMI. Importantly, at baseline, there were no statistically significant differences between groups for any of the variables in Table 2. There were several significant within- and between-group differences at post-intervention. Weight, BMI, waist circumference, and % body fat decreased significantly more in F&S!+ compared to F&S!. Total fat mass decreased significantly within F&S!+ (−4.0%, *p* < 0.001) compared to F&S! (+0.6%, *p* > 0.05) and the difference was statistically significant (*p* < 0.001). The same pattern occurred with VAT mass (−4.1% vs. +3.7%, *p* < 0.05) and VAT volume (−4.1% vs. +3.7%, *p* < 0.05). Percent lean mass increased significantly in F&S!+ but not in F&S!, and the between-group difference was significant (1.5% vs. 0.2%, respectively; *p* < 0.001). However, absolute lean mass did not change over time in either group. Attendance was similar between groups, with F&S! attending on average 70% of classes and F&S!+ attending 64% of classes.

Table 3 shows no statistically significant differences at baseline between groups for any variable listed. Fasting serum insulin and HOMA-IR decreased significantly in F&S!+ (−13.9% and −17.3%, respectively; *p* < 0.05 for both) but not in F&S!; however, there was no significant between-group difference.

Table 4 shows that the change in CRP post-intervention was significantly positively correlated with change in VAT mass, independent of change in weight (*r* = 0.33, *p* = 0.0006). Change in total fat mass was significantly positively correlated with change in fasting glucose, independent of change in weight (*r* = 0.29, *p* = 0.003). Likewise, change in total fat mass was also correlated with change in CRP but, after Bonferroni correction for multiple testing, the correlation was no longer significant (*r* = 0.27, *p* = 0.050).

## 4. Discussion

Our results show that an 8 week (24 session) PA plus weight loss intervention (F&S!+) in older, overweight and obese AAs with osteoarthritis can produce decreases in body fat and VAT that are significantly greater than the changes produced by the PA intervention (F&S!) alone. This finding is consistent with previous studies comparing the effects of PA versus PA plus weight loss on body fat and body fat distribution in older adults [37,38,47,48,49]. However, unlike previous studies, our findings are from a primarily African American cohort. Contrary to our hypothesis, the F&S!+ intervention was not superior in regard to improving inflammation or glucose metabolism-related biomarkers compared to F&S!. Nonetheless, within groups, we saw a significant reduction in fasting serum insulin and HOMA-IR in FNS!+, and a significant reduction in TNF-α in the F&S! group post-intervention.

Studies examining the effect of PA and PA plus weight loss on inflammation and glucose metabolism-related biomarkers in older, overweight and obese adults is somewhat mixed. We surmise that the lack of effect in our study may be a result of the short intervention period and relatively low-intensity PA overall and modest decreases in total fat mass (4.0%) and VAT mass (4.1%) in the F&S!+ group. Total fat mass and VAT mass have been shown to be related to IL-6, CRP, and TNF-α [19,23,24] and glucose metabolism-related biomarkers [65]. In other studies measuring and reporting total fat mass and VAT mass, the PA plus weight loss groups achieved at least a 10% decrease in total fat mass [42,47,49] and/or VAT fat mass [37,38,42]. The greater decreases in total fat and VAT mass in these studies are likely due in part to the longer duration of the interventions, with most lasting at least 1 year. Thus, in our study, a longer intervention may have led to greater between-group differences for total fat and VAT mass that would have translated to significant improvement in pro-inflammatory and glucose metabolism-related biomarkers. The relatively small sample size in our study may have also precluded our ability to observe significant between-group effects given studies reporting significant results had at least 100 subjects per treatment arm [36,38].

Our within-group effects on glucose metabolism and pro-inflammatory-related biomarkers were encouraging. For example, fasting serum insulin and HOMA-IR decreased significantly from baseline in the F&S!+ group. The decrease in HOMA-IR is important given the cut-off value for defining insulin resistance using HOMA-IR is 2.6 and the F&S!+ group fell below this cut off, suggesting that the intervention was successful in ameliorating insulin resistance [66]. Surprisingly, TNF-α decreased significantly in F&S! but not the F&S!+ group although the magnitude of decrease was similar in both groups. This result may merely be an experimental artefact and not indicative of a true, underlying biological mechanism given F&S!+ had the same amount of PA and, over time, saw significant decreases in overall and VAT fat mass. Moreover, attendance at group sessions and physical activity participation was similar between the groups meaning differential adherence to the intervention protocol does not explain the difference [67]. The effects of PA and PA plus weight loss on TNF-α in older adults have varied. Tomeleri and colleagues reported that 8 weeks of resistance exercise training in obese, older women was associated with significantly lower TNF-α post-intervention compared to controls [50]. However, Nicklas and colleagues reported no statistically significant decrease in TNF-α in older, overweight and obese women following an 18 month PA plus weight loss intervention [41]. Nicklas suggested that lack of decrease in TNF-α may be due to the protein’s transient production and short half-life. Moreover, Friedenreich and colleagues reported no change in TNF-α following a 12 month aerobic exercise intervention among post-menopausal women [39]. Both Nicklas and Friedenreich suggested that TNF-soluble receptors may glean more robust results of the effects of PA and PA plus weight loss on the biological activity of TNF-α given it is a more stable protein [68].

In previous studies, VAT mass was found to be positively associated with pro-inflammatory cytokines such as IL-6 [23,38] and the acute phase protein, CRP [38,39], and increased overall adiposity is positively associated with TNF-α [19,24] and CRP [38,39]. Somewhat consistent with previous literature, our study showed that decreased VAT mass and total fat mass were positively correlated with decreases in CRP. Moreover, not only was there a correlation between decreased CRP and both VAT mass and total fat mass, the correlation existed independently of weight loss. These findings suggest that even if overall weight remains stable during an intervention, but VAT mass and total fat mass is reduced, it can translate to a concurrent reduction in CRP. However, in contrast to CRP, decreased total fat mass and VAT mass was not correlated with changes in IL-6 or TNF-α. It is possible that the null result for IL-6 is related to the only modest changes to the measures of adiposity given up to 25% of IL-6 originates from adipose tissue [69]. The null result for TNF-α may have been due to the transient production and short half-life of circulating TNF-α [41]. However, it is also possible that the interventions caused a local vs. paracrine effect on TNF-α activity given studies suggesting that local adipose expression of TNF-α versus circulating concentrations are affected more with increased adiposity [70]. Nonetheless, Beavers and colleagues, did detect a significant positive correlation between changes in VAT mass and circulating IL-6 in response to weight loss in older overweight and obese adults with OA. The method used to assess VAT was computed tomography (CT), a more accurate tool to measure VAT than DXA. This likely allowed for detection of even subtle changes in VAT mass [38].

This study had several strengths. It recruited a cohort that is underrepresented in the literature and collected a wealth of information, including cancer- and chronic disease-risk biomarkers and detailed body composition measurements. It also examined the effect of an intervention conducted in a community setting, that is, a setting wherein environmental variables, more so than artificial experimental variables, shape participant behavior and health. Furthermore, the interventions are kept low cost and designed to be easily implemented into existing community structures, such as programming within local park districts. This allows for maximal generalizability.

This study did have limitations, however. First, the intervention lasted only 8 weeks, and one-third of the PA sessions consisted of flexibility and balance training, two activities requiring very limited energy expenditure. This means the interventions may not have been long enough or energy-intensive enough to see clinically significant weight loss (3–5%). Additionally, the nutrition aspect of F&S!+ was not implemented by a trained nutritionist or dietitian. This feature may have rendered the intervention less effective, but also increases the likelihood that the intervention can be replicated successfully in the community. Further, though the interventionists were not necessarily credentialed, they were trained for 14 h. Second, insulin resistance was calculated using the HOMA-IR equation. It was not measured with the hyperinsulinemic-euglycemic clamp, which is the gold standard [71]. This limitation is understandable given the considerable resources needed to perform this procedure (e.g., tightly controlled diet and activity in an in-patient setting) [42]. Third, given the comparative effectiveness design, there was no true control group, which would have allowed for a test of the independent effects of PA. Fourth, the sample size was a limitation. Between-group differences in glucose and HOMA-IR were nearly significant and might have reached significance with greater numbers of participants. Furthermore, other studies with similar sample sizes also did not show differences between the PA and PA plus weight loss groups for inflammatory markers [41,72]. Lastly, all of our subjects had self-reported LE OA. Systemic inflammation is higher in this population [41]. Thus, it is possible that our findings are limited to overweight and obese older adults with this condition.

## 5. Conclusions

In conclusion, while PA plus weight loss (F&S!+) did not significantly decrease pro-inflammatory or glucose metabolism-related biomarkers more than PA alone (F&S!) did, it did decrease overall and visceral fat mass, both of which are associated with increased cancer and chronic disease risk. Furthermore, within F&S!+ but not F&S!, fasting serum insulin and HOMA-IR decreased significantly. Due to the relatively uncontrolled conditions in this study and its short duration, the improvements in cancer- and chronic disease-risk biomarkers, though small, suggest that longer trials might have greater benefit for preventing cancer and chronic disease.

## Figures and Tables

**Table 1:** Demographic and health characteristics of participants.

	Fit and Strong!+ (*n* = 72)	Fit and Strong! (*n =* 83)	All (*n =* 155 ^1^)
Age, year, mean (SD)	66.5 (4.8)	67.2 (5.7)	66.9 (5.3)
Aged 60–69 years, *n* (%)	60 (83%)	61 (73%)	121 (78%)
Aged ≥70 years, *n* (%)	12 (17%)	22 (27%)	34 (22%)
Female, *n* (%)	64 (89%)	73 (88%)	137 (88%)
Education, year, mean (SD)	14.3 (1.6)	13.9 (2.1)	14.1 (1.9)
Not HS graduate, *n* (%)	3 (4%)	9 (11%)	12 (8%)
HS graduate/GED, *n* (%)	10 (14%)	9 (11%)	19 (12%)
Some college or tech school, *n* (%)	32 (44%)	38 (46%)	70 (45%)
College graduate, *n* (%)	27 (38%)	27 (33%)	54 (35%)
Retired, *n* (%)	49 (68%)	63 (76%)	112 (72%)
Married, *n* (%)	12 (17%)	17 (20%)	29 (19%)
Income, median	25,000	25,000	25,000
Chronic conditions ^2^, mean (SD)	2.7 (1.5)	3.1 (1.9)	2.9 (1.7)
Type 2 diabetes, *n* (%)	19 (26%)	25 (30%)	44 (28%)
Hypertension, *n* (%)	58 (81%)	66 (80%)	124 (80%)
Heart disease, *n* (%)	8 (11%)	11 (13%)	19 (12%)

^1^*n =* 132 for income. ^2^ Number of self-reported conditions (of 17) currently affecting health.

**Table 2:** Anthropometrics and body composition: adjusted ^1^ means at baseline and post-intervention visits.

	Fit and Strong!+			Fit and Strong!			
	Baseline (*n =* 72) Mean (SE)	Post-Int (*n =* 65) Mean (SE)	Change ^2^ (%)	Baseline (*n =* 83) Mean (SE)	Post-Int (*n =* 74) Mean (SE)	Change ^2^ (%)	*p* ^3^
Weight, kg	95.5 (2.7)	93.6 (2.7)	−1.8 (−1.9%) ***	97.2 (2.3)	97.0 (2.3)	−0.2 (−0.2%)	<0.001
BMI, kg/m ^2^	33.7 (0.9)	33.0 (0.9)	−0.7 (−2.1%) ***	33.9 (0.8)	33.8 (0.8)	−0.1 (−0.2%)	<0.001
Waist, cm	115.3 (1.0)	113.1 (1.0)	−2.2 (−1.9%) **	114.7 (1.0)	114.8 (1.0)	0.0 (0.0%)	0.02
% body fat	41.6 (0.4)	40.7 (0.4)	−0.9 (−2.1%) ***	41.3 (0.4)	41.2 (0.4)	−0.1 (−0.3%)	<0.001
Fat mass, g	41348 (663)	39699 (705)	−1649 (−4.0%) ***	41525 (599)	41282 (631)	−242.6 (−0.6%)	<0.001
% lean mass	55.4 (0.4)	56.2 (0.4)	0.8 (1.5%) ***	55.7 (0.4)	55.8 (0.4)	0.1 (0.2%)	0.001
Lean mass, g	53603 (783)	53394 (783)	−209 (−0.4%)	54577 (629)	54561 (629)	−15.9 (0.0%)	0.45
VAT mas, g	1992 (102)	1910 (118)	−82.6 (−4.1%) *	1987 (85)	2062 (85)	74.2 (3.7%) *	0.003
VAT volume, cm ^3^	2112 (109)	2024 (125)	−87.5 (−4.1%) *	2107 (90)	2185 (91)	78.7 (3.7%) *	0.003

^1^ From GEE models adjusted for gender, age, site, and baseline BMI: E(Y) = α + β1group + β2time + β3group × time + β4gender + β5age + β6site + β7 (baseline BMI). All biomarker variables are log-transformed to improve normality. Ns differ slightly for some biomarker variables.^2^ Test for within-group difference in change from baseline to post-intervention visit: * *p* < 0.05, ** *p* < 0.01, and *** *p* < 0.001. ^3^ Test for difference between groups in change from baseline to post-intervention visit. BMI = body mass index; VAT = visceral adipose tissue.

**Table 3:** Cancer-risk biomarker variables: adjusted ^1^ geometric means at baseline and post-intervention visits.

	Fit and Strong!+			Fit and Strong!			
	Baseline (*n =* 72) Mean (95% CI)	Post-Int (*n =* 65) Mean (95% CI)	Change ^2^ (%)	Baseline (*n =* 83) Mean (95% CI)	Post-Int (*n =* 74) Mean (95% CI)	Change ^2^ (%)	*p* ^3^
CRP, mg/L	4.5 (3.1–6.4)	4.2 (3.0–6.0)	−0.2 (−4.9%)	3.5 (2.5–4.8)	3.4 (2.5–4.7)	−0.1 (−1.8%)	0.83
IL-6, pg/mL	3.6 (3.1–4.3)	3.8 (3.2–4.4)	0.2 (4.3%)	3.2 (2.7–3.7)	3.2 (2.8–3.8)	−0.1 (−1.9%)	0.83
TNF-α, pg/mL	6.9 (4.3–10.9)	4.9 (3.0–8.1)	−2.0 (−28.6%)	5.8 (3.6–9.4)	3.7 (2.2–6.2)	−2.1 (−36.2%) *	0.69
Glucose, mg/dL	104.0 (98.1–110.2)	99.9 (94.2–106.1)	−4.0 (−3.9%)	102.9 (98.0–108.0)	104.4 (98.5–110.6)	1.5 (1.4%)	0.09
Insulin, µIU/mL	11.1 (9.1–13.6)	9.6 (7.7–12.0)	−1.5 (−13.9%) *	9.8 (7.9–12.2)	9.4 (7.7–11.6)	−0.3 (−3.5%)	0.23
HOMA-IR	2.9 (2.3–3.6)	2.4 (1.8–3.0)	−0.5 (−17.3%)	2.5 (2.0–3.2)	2.4 (1.9–3.1)	−0.1 (−2.1%)	0.11

^1^ From GEE models adjusted for gender, age, site, and baseline BMI: E(Y) = α + β1group + β2time + β3group × time + β4gender + β5age + β6site + β7 (baseline BMI). All biomarker variables are log-transformed to improve normality. Ns differ slightly for some biomarker variables. ^2^ Test for within-group difference in change from baseline to post-intervention visit: * *p* < 0.05. ^3^ Test for difference between groups in change from baseline to post-intervention visit. CRP = C-reactive protein, IL = interleukin, TNF-α = tumor necrosis factor-alpha, HOMA-IR = homeostasis model assessment-insulin resistance.

**Table 4:** Partial Spearman rank correlations for changes in cancer-risk biomarkers with changes in VAT mass and total fat mass, adjusted for change in weight.

	Δ Insulin, µIU/mL	Δ Glucose, mg/dL	Δ TNF-α, pg/mL	Δ IL-6, µIU/mL	Δ CRP, mg/L
**Δ**VAT mass, g	*r* = 0.018	*r* = 0.11	*r* = 0.12	*r* = 0.14	*r* = 0.33 ^1^
	*p* = 0.86	*p* = 0.27	*p* = 0.24	*p* = 0.16	*p* = 0.0006
**Δ** Total fat mass, g	*r* = 0.053	*r* = 0.29 ^1^	*r* = 0.11	*r* = 0.16	*r* = 0.27
	*p* = 0.59	*p* = 0.0027	*p* = 0.26	*p* = 0.096	*p* = 0.005

^1^ Significant at *p* < 0.005 (Bonferroni correction for multiple testing: 0.05/10). VAT = visceral adipose tissue, TNF = tumor necrosis factor, IL = interleukin, CRP = C-reactive protein.
